# The role of individual differences in environmental sensitivity in teachers' stress and burnout at work

**DOI:** 10.1002/smi.3491

**Published:** 2024-10-08

**Authors:** A. Sperati, M. E. Persico, R. Palumbo, M. Fasolo, M. Spinelli, M. Pluess, G. D’Urso, F. Lionetti

**Affiliations:** ^1^ Department of Neurosciences, Imaging, and Clinical Sciences University G. d’Annunzio Chieti‐Pescara Italy; ^2^ Department of Psychology University G. d'Annunzio Chieti‐Pescara Italy; ^3^ School of Psychology University of Surrey Guildford UK; ^4^ Department of Law, Economics, and Human Sciences Mediterranean University of Reggio Calabria Calabria Italy; ^5^ Depertment of Nervous System and Behavior Sciences University of Pavia Pavia Italy

**Keywords:** burnout, education, environmental sensitivity, school climate, sensory processing sensitivity, stress, teachers

## Abstract

School teachers are among workers most exposed to stress and burnout—a relevant occupational phenomenon leading to psychological and economic costs. The Environmental Sensitivity individual trait—as captured by the psychological marker of sensory processing sensitivity (SPS)—has been found to have a relevant role in stress and emotional exhaustion at work. Yet, little is still known about heightened SPS in the educational field and on underlying mechanisms occurring in the relationship between SPS, stress and burnout. The current work aimed to explore the association between SPS and burnout among teachers as well as the moderating role of perceived stress and school climate in this association. One hundred and ninety eight teachers (44.3 years; SD = 9.7, 94% F) reported on their levels of SPS, occupational burnout, perceived stress and school climate quality. In line with a vulnerability effect, we found heightened SPS largely associated with burnout. This was particularly evident in a context of high‐perceived stress, suggesting that teachers high on SPS may experience more challenges in the face of elevated stress with the need of more support. When exposed to positive and supportive school climate, highly sensitive teachers showed a decrease in burnout, suggesting high SPS as a valuable strength for benefiting from positive experiences. Findings have the potential to inform the customisation of support programs, assisting both schools and work agencies in increasing their awareness of the role of individual differences in responding to both work‐demand‐related stress and to positive work environments.

## 1 INTRODUCTION

School teaching is one of the most stressful professions with the highest risk of occupational burnout, a psychological phenomenon characterised by an overwhelming exhaustion in response to unmanaged stress (for a review see Madigan et al., 2023; see also empirical work Einav et al., 2024). Likely because of the high and prolonged workday demands at school, including workload and time pressure, managing student behaviours, and working in less than optimal economic conditions, research suggests that school teachers are at a relatively high risk of experiencing stress, and ultimately, burnout symptoms (see meta‐analytic findings Garcìa‐Carmona et al., 2019). This leads to significant psychological costs for the individual, and economic and organisational costs for the society at large.

Beside contextual variables, such as the specific job and work conditions, individual variables are also likely to play a role in the subjective experience of distress and burnout. Recent empirical studies (Golonka & Gulla, 2021; Redfearn et al., 2020; Vieregge et al., 2023) specifically suggest that the individual trait of environmental sensitivity (ES; Pluess, 2015), capturing an increased reactivity and responsivity to internal feelings and environmental experiences, is associated with more frequent negative emotions in less than optimal working environments (Golonka & Gulla, 2021; Redfearn et al., 2020). Yet, in line with a Differential Susceptibility effect (Belsky et al., 2007; Belsky & Pluess, 2009), ES has been also reported to contribute to flourishing when the environment is more supportive and less stressful (Vieregge et al., 2023). Based on this, with the current research study, we aim to provide an empirical contribution in this field by exploring the role of individual differences in ES—as captured by the psychological marker of sensory processing sensitivity (SPS; Aron & Aron, 1997)—in occupational burnout in the educational context. In doing so, we involved a sample of teachers working in primary and secondary schools, taking into account other individual variables, such as self‐perceived stress, and perceived quality of the environment, such as school climate. We expect that school teachers that score high on ES may experience higher levels of emotional distress than less sensitive teachers, and that they may be more likely to score higher on burnout as a result of this increased emotional distress. However, in line with a for better and for worse hypothesis, we also expect that their levels of burnout could decrease if the work context, the school environment in this specific case, is perceived as supportive and overall more positive.

Enhancing our comprehension of how individual differences in SPS contribute to school teachers' stress and burnout has the potential to inform the customisation of prevention and intervention programs. This tailored approach may have positive effects not only for teachers but also for students and the overall school environment.

### 1.1 Burnout among teachers: The role of stress and school climate

Being exposed to work‐related stress that has not been well managed, or to an adverse working climate can lead to symptoms of burnout. The burnout syndrome refers to an occupational and psychological phenomenon that arises in response to work‐related unmanaged stress (Maslach & Leiter, 2016; Salvagioni et al., 2017). It is characterised by overwhelming exhaustion accompanied by a profound sense of depersonalisation and ineffectiveness. The issue of occupational burnout has gained increasing attention due to its significance for individual, organisation health, and the general public at large. Burnout strongly influences job performance, leading to cascading effects and substantial costs. Overall, individuals experiencing burnout undergo a deep professional crisis that involves both the emotional and cognitive domains, resulting in a strong sense of job disengagement and detachment (Maslach et al., 2001; Maslach & Leiter, 2016). Workers experiencing burnout report a range of problems, from physical to mental health issues, including low immune defence (e.g., a higher risk of frequent colds, flu, and headache), sleep difficulties, cardiovascular and musculoskeletal problems as well as clinical depressive symptoms and emotion dysregulation (e.g., frustration, helplessness, and irritable mood). This symptomatology often leads to serious physical and psychological health problems with high costs for the individual, organisations, and work contexts including, for example, absenteeism, turnover, resignations, and career changes (Garcìa‐Carmona et al., 2019; Grayson & Alvarez, 2008; Salvagioni et al., 2017; Swider & Zimmerman, 2010; WHO, 2019). Among different categories of workers, school teachers are particularly prone to experiencing burnout (Madigan et al., 2023), likely due to the high demands they face during the workday. It is currently estimated that burnout is evident in around 30%–40% of the school teacher population, with the most often reported symptoms being fatigue, hyperactivity, annoyance, mistrust, and difficulties in interpersonal relationships, significantly impacting their quality of life (Capel, 1991; García‐Carmona et al., 2019; Hakanen et al., 2006; Schonfeld & Bianchi, 2016). More in‐depth qualitative investigations of stress and burnout related to school teaching (Arvidsson et al., 2019; Mota et al., 2021) showed that what teachers seem to find more distressing is the high workload (including administrative and bureaucratic tasks that interfere with lessons planning), the large classroom dimensions, and subsequent sense of inadequacy, ultimately contributing to stress and burnout. This increase in workload, along with high job control, time pressure and overcrowded classrooms, seem to lead to a feeling of not being able to achieve pedagogical goals, resulting in frustration and stress. Importantly, the effects of burnout extend beyond the individual level and affect teacher‐student relationships, with burned‐out teachers showing less flexibility, lower acceptance, and reduced empathy towards students' needs and behaviours, leading to a negative effect on students' performance and motivation. In the end, this could influence students' future professional growth and their attitude to study (Herman et al., 2018; Klusmann et al., 2016). This call for the need of identifying teachers potentially more at risk, to promptly support them, and for prevention and support programs to strengthen teachers' resilience and wellbeing.

Given the relevance of school teachers' burnout at different levels, empirical evidence has focused on investigating both organisational and individual variables that contribute to the development of burnout (Cano‐Garcia et al., 2005; Jennings & Greenberg, 2009; Leiter & Maslach, 2004; Maslach & Leiter, 2016). Among organisational variables, school climate seems to play a pivotal role in teachers' burnout. Specifically, school teachers exposed to an organisational climate characterised by work overload, time pressure, overcrowded classrooms, students' misbehaviours, and unsupportive relationships with superiors, colleagues and parents were more likely to report feelings of exhaustion and being overwhelmed (Garcìa‐Carmona et al., 2019). Among individual variables, empirical findings provide evidence that teachers' age was negatively associated with burnout, with younger school teachers showing higher emotional exhaustion than older ones (Lau et al., 2005). Moreover, meta‐analytic findings show that the personality trait of neuroticism is positively associated with a greater risk of experiencing burnout symptoms (Roloff et al., 2022), and teachers' self‐perceived stress is also strongly related to burnout symptoms (Abel & Sewell, 1999). If we extend our focus beyond the population of school teachers, additional research indicates that individuals with elevated scores on the trait of SPS are also more susceptible to experiencing stress and burnout in the workplace, as we review in more detail below.

### 1.2 Sensory processing sensitivity in the work context

Approximately a third of individuals from the general population (Lionetti et al., 2018) are more prone to getting easily overwhelmed, as described by the individual trait of ES. A reliable psychological marker of ES is the sensory processing sensitivity trait (SPS, Aron & Aron, 1997), according to which high sensitivity manifests in a heightened sensory sensitivity and increased emotional reactivity. Empirical evidence on the SPS trait has contributed to the expansion of our knowledge on how individuals differ in their responses to a variety of contexts, including the family environment, support programs, and the workplace (Golonka & Gulla, 2021; Pieroni et al., 2023; Redfearn et al., 2020; Vander Elst et al., 2019; Vieregge et al., 2023). Consistent with a ‘for better and for worse’ effect proposed by Differential Susceptibility theory (Belsky et al., 2007; Belsky & Pluess, 2009), heightened SPS confers an increased vulnerability to stress‐eliciting conditions as well as greater responsiveness to supportive environments. For example, empirical studies provided evidence that people scoring high on the SPS trait are more likely to experience distress and suffer more from internalising symptoms (i.e., anxiety, depression) when exposed to negative and less supportive environments (Bakker & Moulding, 2012; Benham, 2006; Booth et al., 2015; Brindle et al., 2015; Gerstenberg, 2012; Liss et al., 2005). At the same time, highly sensitive individuals appear to show also a greater responsiveness to the positive effect of supportive environments (Kibe et al., 2020; Nocentini et al., 2018; Pluess & Belsky, 2013; Pluess & Boniwell, 2015). Research focussing on SPS in the workplace has identified a significant association between SPS and stress and burnout among employees from different organisations (Golonka & Gulla, 2021; Redfearn et al., 2020; Vander Elst et al., 2019), suggesting that SPS represents a vulnerability factor. Overall, highly sensitive workers, for examples in nursing and other job categories (e.g., IT and financial companies, small and big team managers), reported a greater sense of overwhelm than less‐sensitive ones. A qualitative study showed that such overstimulation could be due to an ease of excitation in front of mild sensory stimuli at work (e.g., bright lights in the environment) characterising highly sensitive people (Bas et al., 2021). The sense of overwhelming has been found to result in a chaotic and unmanageable work, a loss of meaning, and subsequent dissatisfaction, detachment, and a desire for change. Moreover, a However, in line with a ‘for better’ effect, two recent studies also showed that highly sensitive employees in various types of industries and organisations benefit more from positive job characteristics and resources, in terms of positive job attitudes and help behaviours at work compared to less sensitive ones (Vander Elst et al., 2019; Vieregge et al., 2023). This suggests that sensitivity not only plays a role in negative but also in positive work‐related feelings and emotions. In this study, we will take this line of research a step further, considering the educational work context which is known for the higher levels of stress identified at work (Garcìa‐Carmona et al., 2019; Madigan et al., 2023). We will do this involving a sample of teachers working in primary and secondary schools, exploring bivariate associations but also potentially interactive patterns between individual and environmental variables.

### 1.3 Overview of the current study

The current study had two aims. First, to explore the effect of individual differences in SPS on school teachers' burnout. Second, to investigate whether perceived stress and the school organisational climate moderated the association between SPS and burnout. We accomplished this aim by exploring variables in a sample of Italian teachers from different regions, surveyed online during March and April 2023. Based on literature on SPS, we expected that increased SPS would predict higher levels of burnout symptoms in school teachers. Individuals high in SPS are more vulnerable to the negative effect of stress‐related and unfavourable conditions, and we anticipate that this association would be stronger in a more stressful context and when teachers were exposed to a less‐than‐optimal school climate. Additionally, given that high SPS has been considered a marker of Environmental Sensitivity (Pluess, 2015), we also expected that higher levels of SPS would predict greater responsiveness to positive and supportive school environments. In other words, we anticipate school teachers scoring high on SPS to benefit more than others of a high‐quality school climate, decreasing their levels of burnout when the environment is perceived as supporting.

## 2 MATERIAL AND METHOD

### 2.1 Participants and procedure

Participants were *N* = 198 Italian school teachers who completed an online survey including measures on SPS, burnout, stress and school climate. Teachers' mean age was 44.3 years (SD = 9.7) and 94% were female (this gender percentage aligns with the general teacher population in Italy, see Angelini et al., 2021). In terms of educational levels, 52% had a master degree, 24% had a post‐lauream degree (i.e., post‐lauream master courses and/or PhD degree) whereas 17% of teachers had a high school degree. Most of the teachers, 38%, taught in primary school, followed by 27% of teachers who worked in secondary school, 19% in middle school and 10% in kindergartens. The majority, that is, 64%, had a permanent contract, 63% were tenured teachers, and 27% were substitute teachers. The sample was recruited from different areas in Northern (32%), Southern (10%), and Central Italy (51%) to reach a sample as representative as possible, following a snowball procedure. Recruitment mainly occurred via social media, including posting the study in various teacher groups and sharing the survey through personal contacts. Our aim was to recruit a sample size as big as possible given a specific window of time. The research team directly reached out to groups and contacts, encouraging them to share the link among their colleagues. Informed consent was obtained from all participants who were informed about the study conditions and invited to join an informative seminar at the study's conclusion. Participants were also provided with email addresses of person responsible of the study in case they needed to contact them for support. All data are openly available at the following link https://gitfront.io/r/user‐8766068/T6kFct6zPjMD/SPS‐and‐teachers‐stress‐and‐burnout/.

### 2.2 Measures

#### 2.2.1 Sensory processing sensitivity

SPS levels were measured using the 12‐item Highly Sensitive Person scale (Pluess et al., 2023), recently validated in an Italian population (Lionetti et al., 2024). The HSP‐12 aims at capturing a strong feeling of getting overwhelmed (e.g., ‘Do changes in your life shake you up?’, ‘Do you get rattled when you have a lot to do in a short amount of time?’), a low sensory threshold (‘Are you bothered by intense stimuli, like loud noises or chaotic scenes?’) as well as an increased appreciation of and greater attention to subtleties and positive aspects in the surroundings (e.g., ‘Do you seem to be aware of subtleties in your environment?’; ‘Do you notice and enjoy delicate or fine scents, tastes, sounds, works of art?’). Each item is rated on a seven‐point Likert scale ranging from 1 = Not at all to 7 = Extremely, with higher scores indicating higher levels of SPS. The measure provides a total score of general sensitivity. In the current sample, internal consistency of the total score was good (Cronbach's *α* = 0.86) and in line with the validation studies (Lionetti et al., 2024; Pluess et al., 2023).

#### 2.2.2 Burnout

Teachers' burnout was assessed using the Italian validated version of the BAT‐12 (Mazzetti et al., 2022; Schaufeli et al., 2020) that aims at capturing the four core burnout symptoms. Specifically, the 12 items investigate exhaustion (e.g., ‘At work, I feel mentally exhausted’), mental distance (e.g., ‘I struggle to find any enthusiasm for my work’), cognitive impairment (e.g., ‘At work, I have problems staying focused’), and emotional impairment (e.g., ‘At work, I feel unable to control my emotions’). Each item is rated on a 5‐point Likert scale ranging from 1 = Never to 5 = Always, with higher scores indicating higher frequency of burnout symptoms. The measure provides an overall burnout score. In the current sample, internal consistency of the total score was good (Cronbach's *α* = 0.89) and in line with the validation studies (Mazzetti et al., 2022; Schaufeli et al., 2020).

#### 2.2.3 Perceived stress

Teachers' perceived stress was measured with the perceived stress scale (Cohen et al., 1983; Mondo et al., 2021) consisting of 10 items that aim at capturing self‐perceived stress levels. Specifically, items assessed the degree to which people perceive life as unpredictable, uncontrollable and overloading, asking about frequency of feelings and thoughts over the previous month (e.g., ‘In the last month, how often have you felt nervous and stressed’; ‘In the last month, how often have you felt that you were unable to control the important things in your life’). Each item is rated on a 5‐point Likert scale ranging from 0 = Never to 4 = Very Often. The measure provides a total score of individual stress, with higher scores indicating higher levels of perceived stress. In the current sample, internal consistency of the total score was good (Cronbach's *α* = 0.90) and comparable with the validation studies (Mondo et al., 2021).

#### 2.2.4 School climate

Teachers' perception of the quality of their own school climate were assessed using the School Organisational Health Questionnaires in its Italian version (Guidetti et al., 2015; Hart et al., 2000). We considered items belonging to four dimensions that capture the teachers' perception of school morale (e.g., ‘There is good team spirit in this school’), appraisal and recognition (e.g., ‘Teachers receive recognition for good work’), professional interaction (e.g., ‘Teachers in this school can rely on their colleagues for support and assistance when needed’), and supportive leadership (e.g., ‘The administration in this school can be relied upon when things get tough’). Items asked to what extent the statement applies to one's own school and are rated on a 4‐point Likert scale ranging from 1 = Strongly disagree to 4 = Strongly agree. Higher total scores indicate high‐quality school organisational climate. In the current sample, internal consistency of the total score was good (Cronbach's *α* = 0.94) and in line with the validation studies (Guidetti et al., 2015).

### 2.3 Data analysis

We first explored the percentage of missing values and computed bivariate associations between all study variables to investigate whether SPS, burnout, perceived stress and school climate were associated with each other. We considered associations to be low when Pearson's *r* was around 0.10 or less, medium if *r* varied around 0.30, and large if *r* was higher than 0.50 (Cohen, 1988, 1992). Next, to explore the direct effect of SPS on teachers' burnout, we ran a model considering SPS as a predictor of burnout. Subsequently, we conducted a series of main effect models, adding to SPS perceived stress and school climate as predictor variables. Lastly, we performed a series of two‐way interaction models, including perceived stress and climate school as moderating variables (in separate models), to investigate whether SPS predicts teacher burnout depending on levels of perceived stress and the quality of school climate. Considering that the type of school in which teachers worked could influence the school climate and its quality perception, we also ran main effect and three‐way interaction models considering, alongside the school climate, the grade of school (1 = primary school, including teachers working in kindergartens and primary schools, 2 = secondary school, including teachers working in middle and secondary schools). With the three‐way interaction model, we explored whether working in different school grades impacted the extent to which SPS interacts with the school climate in predicting teacher's burnout. In order to evaluate whether the inclusion of the interaction terms improved capability of the models to predict data better, we compared the main effects with the interaction models using the *R* ^2^ (i.e., the total variance of the outcome variable accounted by the model), the AIC (Akaike, 1974), and Akaike weights (Burnham & Anderson, 2002); the log‐likelihood and the *χ* ^2^ test (Satorra, 2000). According to AIC and log‐likelihood the lower the value the better the model is at predicting data, while for *R* ^2^ and Akaike weights, ranging from 0 to 1, the higher the value, the better the model is at describing data accurately (McElreath, 2016; Vandekerckhove et al., 2015; Wagenmakers & Farrell, 2004). Finally, after selecting the best fitting model, we followed up interaction effects by adopting conditional plots. All analyses were run using the statistical software R (R Core Team, 2020).

## 3 RESULTS

### 3.1 Descriptive statistics and bivariate associations among variables

Because the percentage of missing values in the total sample was below 10% (7.3%), we adopted listwise deletion for handling missing data. Descriptive statistics and bivariate associations among all variables are reported in Table 1. Overall, the SPS distribution in our sample was slightly left‐skewed, with the mean value one point higher than that of the general population (mean in the current sample = 5.30, range = 2.5–7, SD = 0.98; mean of the general population = 4.92, SD = 0.80, Lionetti et al., 2024). The distribution of burnout levels and stress are comparable to the general population (De Hert, 2020; Lindblom et al., 2006). Bivariate associations showed that SPS was largely and positively associated with burnout symptoms (*r* = 0.53) and with perceived stress (*r* = 0.54) but negatively and moderately with school organisational climate (*r* = −0.30). SPS was slightly negatively associated with age (*r* = −0.13) but no with gender (*r* = −0.02). Burnout symptoms were largely and positively associated with perceived stress (*r* = 0.67) and negatively with school climate (*r* = −0.36). Associations between burnout and gender and age were close to zero. Variable distribution and bivariate associations including SPS subscales are reported in the Supplementary Material document (Figure S1).

**TABLE 1:** Bivariate associations among all study variables (*N* = 172).

	Mean (SD)	1	2	3	4	5	6	7
1 SPS	5.30 (0.98)	—						
2 Burnout	2.20 (0.63)	0.56	—					
3 Perceived tress	2.13 (0.72)	0.56	0.67	—				
4 School climate	2.41 (0.64)	−0.33	−0.36	−0.26	—			
5 Gender		−0.02	0.04	0.03	−0.02	—		
6 Age		−0.13	−0.05	−0.20	−0.20	0.08	—	
7 School grade		−0.02	−0.13	0.00	0.17	−0.18	−0.14	—

*Note*: Given the sample size, *N* = 172, correlation values greater than 0.15 are significantly different from zero. According to Cohen (Cohen, 1988, 1992): Trivial associations: *r* lower than *r* = 0.10; moderate associations: *r* = 25–45; strong association: *r* equal to or higher than 0.50.

Abbreviation: SPS, sensory processing sensitivity.

### 3.2 Sensory processing sensitivity, perceived stress and their interaction in predicting burnout

**FIGURE 1:**
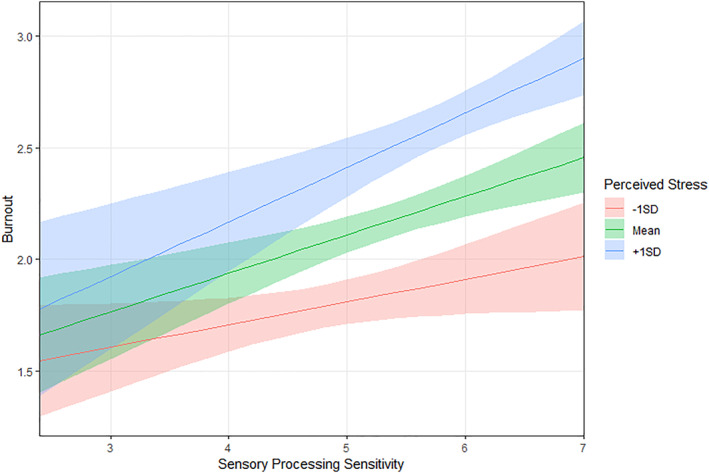
Conditional interaction plot. Each line represents the relation between SPS and burnout conditioned to low (−1SD) medium and high levels (+1SD) of perceived stress. Bands represent the uncertainty of estimates (*N* = 172). SPS, sensory processing sensitivity.

Models including only main effects suggested that both SPS and perceived stress were significantly and positively associated with burnout (*β* = 0.25, *p* = <0.001; *β* = 0.51, *p* = <0.001, respectively). We then tested whether the perceived stress moderated the extent to which SPS predicted burnout. When the interaction term was added to the regression model, *R* ^2^ and Akaike weight values increased, the AIC, LogLik decreased, and supported by the *χ* ^2^, findings suggest the interaction model outperforming the main effect in predicting data better (see Table 2 for results of model comparison). Specifically, SPS was significantly associated with perceived stress in predicting burnout (*β* = 0.82; *p* = 0.03). To interpret the significant interaction, we followed up the relationship between SPS and burnout by plotting simple slopes for low (−1 SD), medium, and high (+1 SD) levels of perceived stress (see Figure 1). The plot suggested that teachers high on SPS trait reported significantly higher levels of burnout symptoms than less‐sensitive ones when the perceived stress was high. For lower levels of stress, burnout was weaker than it was when stress was high in teachers high on SPS. For lower scores in SPS, the increase of burnout when stress increased was much weaker. As a follow‐up test, we run a simple slope analysis. Findings suggested that the association between SPS and burnout was significant at all levels of SPS but with a continuous gradient effect. Particularly, when stress was high (+1SD), the effect of SPS on burnout was *B* = 0.24 (0.06), *p* < 0.001, for medium level of stress it decreased at *B* = 0.17 (0.04), *p* < 0.001, and for low levels of stress (‐1SD), the effect was trivial and equal to *B* = 0.10 (0.05), *p* = 0.044.

**TABLE 2:** Comparison of regression models considering SPS and perceived stress in predicting burnout.

Models	*R* ^2^	AIC	Delta	Akaike weights	LogLik	*χ* ^2^	*p χ* ^2^
Model 3 (SPS × perceived stress)	**0.48**	**223**	**0.00**	**0.79**	**−106**	**4.76**	**<0.02**
Model 2 (SPS + perceived stress)	0.47	226	2.6	0.21	−108	52.14	<0.001
Model 1 (SPS)	0.29	277	63.8	0.00	−134		

*Note*: Bold values represent the best model in predicting data.

Abbreviations: LogLik, log‐likelihood; SPS, sensory processing sensitivity.

Regression assumptions were met, as suggested by the follow‐up residual plots showing that residuals were approximately normally distributed (see Figure S2 in Supplementary Material).

### 3.3 Sensory processing sensitivity, school climate and school grade in predicting burnout

**FIGURE 2:**
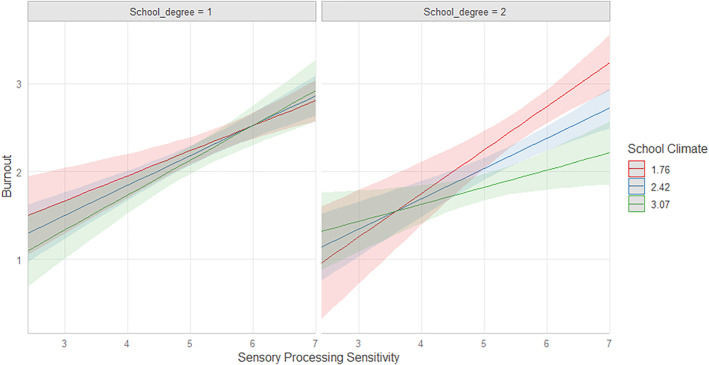
Three‐way interaction plot. Each line represents the relation between SPS and burnout conditioned to low (−1SD) medium and high levels (+1SD) of school climate among teachers teaching working in primary schools (school degree = 1 on the left) and teachers working in secondary school grade (school degree = 2 on the right). Bands represent the uncertainty of estimates (*N* = 172). SPS, sensory processing sensitivity.

We first ran a series of main effect models including SPS, school climate and school grade as predictor variables. Results suggested that SPS was significantly and positively associated with burnout (*β* = 0.50, *p* = <0.001), school climate was significantly and negatively associated with burnout (*β* = −0.22, *p* = 0.001), but school grade (teaching in primary schools vs. secondary schools) was not meaningfully associated with burnout (*β* = −0.10, *p* = 0.43). We then ran a series of two‐way interaction models, considering SPS × school climate and SPS × school grade as separated models, and no significant interaction effects were found (*β* = −0.13, *p* = 0.69, *β* = 0.27, *p* = 0.50, respectively). When the three‐way interaction term was added (i.e., SPS × school climate × school grade), the increase of *R* ^2^ and the Akaike weights, and the decrease of AIC and LogLik criteria suggested that the model outperformed both main and two‐way interaction effects in predicting data better (see Table 3), and a significant effect was found. Precisely, SPS was significantly associated with school climate and school grade in predicting burnout (*β* = −4.36, *p* = 0.01). To interpret the significant three‐way interaction, we plotted the relationship between SPS and burnout with simple slopes conditioned to low (−1 SD), medium, and high (+1 SD) levels of school climate quality and to the school grade (1 = Primary school; 2 = Secondary school). The follow‐up plot showed that the relationship between SPS and burnout was moderated by the quality of the school climate, and this was evident for teachers working in secondary school, but not in primary school, where SPS was positively associated with burnout regardless the quality of school climate (see Figure 2). Specifically, when the school climate was low, teachers scoring high on SPS and working in secondary schools showed the highest levels of burnout but when they experienced a positive school climate the relationship weakened, and their levels of burnout decreased, though they remained higher than those of teachers with lower SPS scores. Conversely, less‐sensitive teachers showed overall low levels of burnout regardless the quality of the school climate. As a follow‐up test, we run a simple slope analysis suggesting that the association between SPS and burnout was significant at all levels of school climate quality—low, medium and high ‐ with a similar effect (*B* = 0.38 (0.06), *p* < 0.001, *B* = 0.34 (0.04), *p* < 0.001, *B* = 0.30 (0.06), *p* < 0.001, respectively).

**TABLE 3:** Comparison of regression models considering SPS, school climate and school grade in predicting burnout.

Models	*R* ^2^	AIC	Delta	Akaike weights	logLik	*χ* ^2^	*p χ* ^2^
Model 7 (SPS × school climate × school grade)	**0.42**	**254**	**0.0**	**0.83**	**−117**	**14.2**	**<0.001**
Model 6 (SPS + school climate + school grade)	0.37	260	5.5	0.05	−125	9.2	<0.001
Model 5 (SPS × school grade)	0.34	269	14.7	0.00	−130	0.5	0.49
Model 4 (SPS + school grade)	0.34	268	13.1	0.00	−130	8.6	<0.001
Model 3 (SPS × school climate)	0.34	261	6.6	0.03	−126	0.2	0.69
Model 2 (SPS + school climate)	0.37	259	4.6	0.08	−126	10.1	<0.001
Model 1 (SPS)	0.33	268	13.5	0.00	−131		

*Note*: Bold values represent the best model in predicting data.

Abbreviation: SPS, sensory processing sensitivity.

## 4 DISCUSSION

Because of the high demands of their daily work, teachers are among the workers most exposed to stress, leading to an increased risk of occupational burnout and consequential economic and psychological costs. In line with the Environmental Sensitivity meta‐framework, a sizeable group of individuals, specifically those scoring high on the basic trait of Sensory Processing Sensitivity (SPS; Aron & Aron, 1997), are likely to experience more often distress and negative affect (SPS; Aron & Aron, 1997; Lionetti et al., 2019), especially when exposed to adverse conditions. Similarly, they are more responsive to supportive experiences. The role of SPS in stress and burnout has been identified also in the workplace. With the current work, we aimed to expand research in the field a step further, by exploring the relationship between SPS and burnout in the educational work context, as well as, by examining the extent to which this association could be moderated by both individual (i.e., perceived stress) and contextual variables (i.e., perceived school organisational and relational climate). Teachers reported on their levels of SPS, occupational burnout, perceived stress and the quality of their school organisational and relational climate. Interestingly, the mean SPS value in our sample of teachers was around one point higher than that found in the general Italian population (Lionetti et al., 2024). This higher average SPS level among teacher population potentially suggests that highly sensitive individuals may be more predispose to look for meaningful professions, particularly in education and helping roles. Additionally, the greater empathy characteristic of highly sensitive people could influence their choice of an educational profession, which implies relationships based on care and growth (Acevedo et al., 2017; Aron et al., 2012; Black & Kern, 2020). This result could also due to the fact that highly sensitive teacher are more likely to be stressed and the higher average SPS could be driven by higher scores in items related to the ease of excitation SPS aspect. Alternatively, because we did not know how many teachers we have been able to reach out, we can also hypothesise that highly sensitive teachers were more interested in taking part in research on these topics (as SPS was found before to be more associated) with burnout in other work contexts (Golonka & Gulla, 2021; Redfearn et al., 2020; Vander Elst et al., 2019), compared to low sensitive ones.

### 4.1 Sensory processing sensitivity, perceived stress, and burnout

As anticipated, we found that SPS was largely associated with occupational burnout and perceived stress, suggesting, at a first glance, that heightened sensitivity is a vulnerability factor. More in detail, it seems that highly sensitive teachers, likely due to their ease of excitation, are more prone to be activated by workday demands and more likely to express negative overwhelming feelings related to work (i.e., emotional exhaustion and mental distance). Such a vulnerability facet of SPS in the workplace has been suggested in previous studies, which showed that high SPS was strongly associated with emotional exhaustion and work disengagement among nurses and other employees (Golonka & Gulla, 2021; Redfearn et al., 2020; Vander Elst et al., 2019). This further aligns with findings in adults from the general population, showing that high SPS was strongly associated with internalising symptoms such as anxiety and depression, especially when exposed to unfavourable experiences (Liss et al., 2005). Moreover, we found that the association between SPS and burnout was stronger when perceived stress was higher, indicating that highly sensitive teachers suffer more when exposed to stress. For less‐sensitive teachers, burnout levels remained low across all levels of stress. In other words, while teachers scoring low in SPS did not experience significant emotional and cognitive exhaustion even in the face of stress, highly sensitive teachers appeared to get overwhelmed by elevated perceived stress. This could be because heightened stress is so challenging to regulate for highly sensitive teachers that it leads to overwhelming feelings, and thus, burnout symptoms, as a response to unmanaged stress. Importantly, from an applied point of view, this finding sheds light on mechanisms underlying the previously found associations between SPS and work‐related emotional exhaustion. It suggests that people differ in their responses to stress with some teachers, specifically those high in SPS, being more prone to burnout due to difficulties in facing individual stress and more in need of support. Given that teachers are frequently exposed to stressful demands in their workday, these findings suggest the relevant need for planning and implementing actions specifically designed for highly sensitive teachers, with potential benefits for all teachers.

### 4.2 Sensory processing sensitivity, school climate, and burnout

Interestingly, when considering the quality of school climate, we found that as the SPS increased, the perceived school climate decreased. Highly sensitive teachers, due to their greater attention and awareness of subtleties in their surrounding, may notice negative or unsatisfactory aspects in the school environment more readily than less‐sensitive ones. Alternatively, this association may be explained by other variables related to SPS, as neuroticism (which do not overlap fully with SPS still it correlates to some extent, Lionetti et al., 2024), that we did not control for as this variable was not administered in the survey. Previous studies proved incremental validity of SPS over personality traits in predicting wellbeing and psychological adjustment (Lionetti et al., 2024), but future research could contribute further to this, potentially providing additional support to this incremental effect.

In addition, we identified that teachers with higher levels of SPS that are serving in secondary school grade, but not in primary schools, tend to benefit from a supportive school climate, though they still remained at a higher risk of burnout symptoms compared to less‐sensitive ones. In other words, when the work environment is characterised by good team morale, positive relationships among colleagues and superiors, and supportive feedback and interactions, burnout levels of highly sensitive teachers decreased compared to when they were exposed to a low‐quality school climate. Contrary to our expectations, we did not find an advantage effect and values of burnout still remained significant, suggesting the need of extra support for highly sensitive teachers, at least in our sample of Italian school teachers. Yet, importantly, we highlight the decrease in burnout. This effect of school climate on the association between SPS and burnout was observed specifically in teachers in secondary school grades, indicating a potential buffering role of positive and supportive school morale for burnout in highly sensitive teachers working with older students. This is likely due to the fact that teachers in higher school grades need to address challenges that could potentially be resolved through a strong network with colleagues who share the same context. Consequently, these findings suggest that activities to strengthen relationships among colleagues and superiors could have a protective role particularly for highly sensitive teachers. In contrast, positive relationships among colleagues did not have an effect on primary school teachers' burnout. This might be because teachers working with younger students potentially encounter concerns that require a different form of support, such as fostering a positive school‐family relationship. However, the school climate, as assessed in the current study, did not specifically consider variables related to teacher‐parent relationships as well as school‐family communications. Future studies should further investigate the potential role of such variables in buffering the effect of SPS on burnout among teachers.

From an applied perspective, existing evidence‐based initiatives for school teachers well‐being address individual (i.e., mitigating the impact of stress, promoting mindfulness mind‐set and socio‐emotional aspects) and contextual (i.e., school organisational climate and demands) dimensions separately, showing scarce to moderate efficacy (for a review see Berger et al., 2022). This overall unsatisfactory efficacy could be due to the fact that most of the initiatives are individual‐based, overlooking context‐related aspects. Moreover, the efficacy of interventions could be underestimated when individual differences in responding to the environment are not considered (Bakermans‐Kranenburg & Van Ijzendoorn, 2015). Drawing from the awareness that some people, due to differences in SPS levels, react more strongly to internal feelings but also may benefit from supportive experiences, schools could adopt intervention and promotional programs that take into account both individual and contextual dimensions. These programs should specifically target highly sensitive teachers but could benefit all teachers, especially during high‐stressful period. For instance, efforts to support teachers in recognising, coping with, and better managing their emotions could be useful, along with providing listening points for teachers at school and allowing them to take breaks when they feel excessively stressed. Moreover, reducing workload could help highly sensitive teachers avoid overstimulation.

From a broader organisational perspective, findings from this study may inform business agencies about individual differences in regards to work demand‐related stress. Being aware of the role of individual characteristics might allow to create a work context as optimal as possible for individuals with different needs, yielding several benefits for job performance (e.g., greater productivity, increased motivation).

## 5 STRENGTHS AND LIMITATIONS

To the best of our knowledge, this is the first study focussing on SPS in the educational work context, investigating the role of SPS, perceived stress, and school climate in predicting teachers' occupational burnout, a noteworthy phenomenon with significant psychological cost for individuals and economic costs for organisations and society at large. Overall, findings provide empirical evidence that teachers scoring high in SPS are more likely to get easily overwhelmed, especially when experiencing high perceived stress, resulting in higher levels of burnout symptoms as a potential response of unmanaged stress, but they are also more able to benefit from high‐quality school environment compared to their less‐sensitive counterpart. However, these findings should be considered in light of some limitations. First, our data were based on online self‐report questionnaires, and teachers' reports on their perception of the environmental variables could be biased through their own lens and mood (e.g., negative feelings or tiredness while filling out the survey as well as isolated negative events that could happen in school). Moreover, levels of teachers' stress could fluctuate during the school year, impacting teachers' burnout differently. Further research should investigate the role of SPS in burnout among teachers more deeply by including observational measures of the school organisational and relational climate, as well as adopting longitudinal designs that allow monitoring teachers' negative feelings and emotional exhaustion. In addition, questionnaires we adopted may also include some items that are similar in contents, and this may have inflated correlations (e.g., getting overwhelmed in front of sudden changes is something captured both by SPS and stress questionnaire). Future work may consider adopting SPS measures that are less biased by negative emotionality. Second, our study lacks measures investigating some individual variables that could play a protective role (i.e., personal attitudes and competencies). Future studies should offer a more comprehensive understanding by considering, for example, the moderating role of individual job attitudes and work engagement in teacher population. In addition, future research could contribute to support further the incremental effect of SPS over other personality traits (e.g., neuroticism) in predicting individual psychological wellbeing. Additionally, exploring whether teacher's individual skills as for example, emotion regulation competencies might have a buffering effect in the association between SPS, stress and burnout would address current knowledge gaps. Lastly, the measure for school climate used in the current study did not specifically consider variables related to teacher‐parent relationships as well as school‐family communications. Future studies should address such variables and further investigate their potential role in buffering the effect of SPS on burnout among primary school teachers. Importantly, our sample was predominantly composed of women, and previous studies have shown that working women tend to experience more exhaustion, likely due to an overlapping of roles (i.e., worker, mother and house care; Golonka & Gulla, 2021). Nevertheless, our results are expected and in line with demographic data among teachers in the Italian school system, where the majority are women (Angelini et al., 2021). Finally, our sample was self‐selected, and it would be interesting to explore if these findings are replicated on bigger and independent samples.

## 6 CONCLUSION

In the current study, we focused on the association between SPS and burnout in the teaching profession—a working field characterised by high stress levels and increased susceptibility to burnout. Moving beyond bivariate associations, we further explored potential interaction patterns investigating the moderating role of perceived stress and school organisational climate in this relationship. Heightened SPS was largely associated with burnout, and this association was particularly evident in a context of high perceived stress, suggesting that highly sensitive teachers may experience more challenges in the face of elevated stress, likely due to their tendency of getting overwhelmed. These findings shed light on potential mechanisms underlying the previously found associations between SPS and emotional exhaustion among both the general population and other categories of workers, such as nurses and employees in different organisations and companies. Highly sensitive teachers might be more in need of support programs, especially for facing with their stress and exhaustion‐related feelings. However, our study suggests that being a highly sensitive individual helps secondary‐school teachers to benefit from a positive and supportive school climate, resulting in decreased burnout levels compared to when exposed to a less‐than‐optimal work environment. Finally, our study addressed the relevant phenomenon of burnout in teaching profession with potential implication for educational and the broad work context at large. Overall, developing supportive actions and programs that focus on the understanding and the management of emotions could be crucial for helping highly sensitive workers in better coping with their responses to work‐related stress. Moreover, our work could inform and assist agencies in the organisational field in increasing their awareness of the role of individual differences in responding to both work‐demand‐related stress and to positive work environments, factors that can significantly impact job performance and society welfare at large.

## AUTHOR CONTRIBUTIONS

Conceptualisation: Alessandra Sperati, Francesca Lionetti, Giulio D’Urso Methodology: Alessandra Sperati Formal analysis and investigation: Alessandra Sperati Writing—original draft preparation: Alessandra Sperati, Melba Emilia Persico Writing—review and editing: Francesca Lionetti, Michael Pluess, Giulio D’Urso, Mirco Fasolo, Maria Spinelli, Riccardo Palumbo. Supervision: Francesca Lionetti, Michael Pluess. All authors read and approved the final manuscript.

## CONFLICT OF INTEREST STATEMENT

The authors have no relevant financial or non‐financial interests to disclose.

## ETHICS STATEMENT

Approval was obtained from the ethics committee of Department of Neuroscience, Imaging and Clinical Sciences, University G.d’Annunzio, Chieti‐Pescara, Italy. The procedures used in this study adhere to the tenets of the Declaration of Helsinki.

## CONSENT

Informed consent was obtained from each participants.

## Supporting information

Supporting Information S1

## Data Availability

The data that support the findings of this study are openly available in gitfront at https://gitfront.io/r/user‐8766068/T6kFct6zPjMD/SPS‐and‐teachers‐stress‐and‐burnout/.
