# Supplementary material for: The role of individual differences in environmental sensitivity in teachers' stress and burnout at work
Source: Stress Health. 2024 Oct 8;40(6):e3491. doi: 10.1002/smi.3491 (PMC11636432; doi:10.1002/smi.3491)

**The role of individual differences in Environmental Sensitivity in teacher’s stress and burnout at work**

**Supplementary material**

19/02/2024

Contents

- 1. Variable distribution……………………………………………………………. 2
  2. Bivariate associations including subscales (N = 172) 3
  3. Residual plots………………………………………………… ………………….… 5

Used R packages […………………………………………………………………..](#_TOC_250000) 8

**Figure S1**. Variable distribution


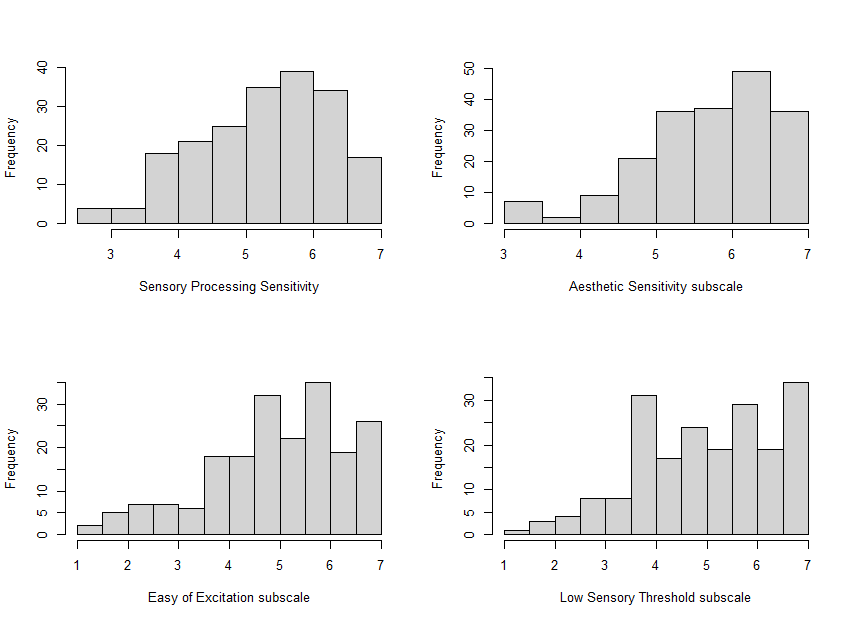

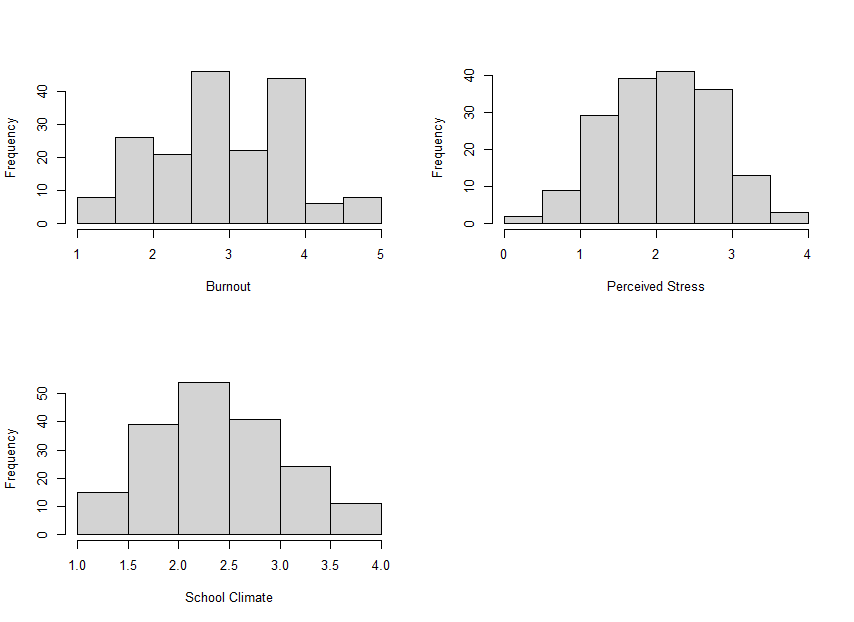


**Table S1.** Bivariate associations between all variables considering SPS subscales

|  | 1 | 2 | 3 | 4 | 5 | 6 | 7 |
| --- | --- | --- | --- | --- | --- | --- | --- |
| 1 SPS | - |  |  |  |  |  |  |
| 2 EOE | .89 | - |  |  |  |  |  |
| 3 AES | .66 | .35 | - |  |  |  |  |
| 4 LST | .83 | .61 | .45 | - |  |  |  |
| 5 School Climate | -.30 | -.27 | -.16 | -.29 | - |  |  |
| 6 Burnout | .53 | .54 | .19 | .46 | -.36 | - |  |
| 7 Stress | .54 | .55 | .22 | .45 | -.25 | .65 | - |

*Note.* SPS = Sensory Processing Sensitivity; EOE = Easy of Excitation; AES = Aesthetic Sensitivity; LST = Low Sensory Threshold

**In Figure S2** are reported residual plots for testing the regression assumption pertaining to the interaction model considering SPS * Stress in predicting burnout. Results suggested that residuals were approximately normally distributed, supporting the regression assumption.

**Figure S2**. Residual plots for testing regression assumption


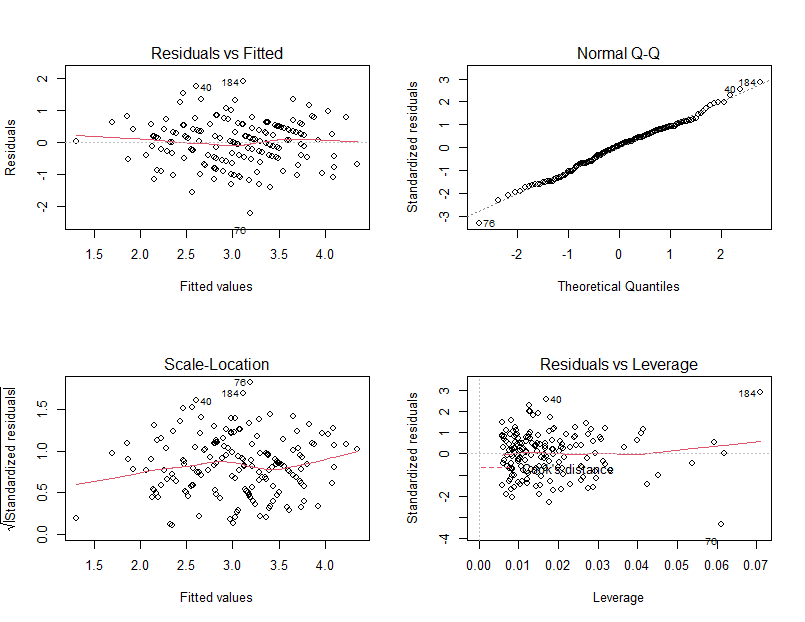

Supplement: Supplementary file 1 — Supporting Information S1 [file SMI-40-e3491-s001.docx]
